# Exploring the Dorian Gray Trait: Unveiling the Complexities of Perceived Aging and Self-Image

**DOI:** 10.7759/cureus.47326

**Published:** 2023-10-19

**Authors:** Sujata Mehta Ambalal, Varun Monga, Rahul Kashyap

**Affiliations:** 1 Dermatology, Skin Clinic at Sumeru, Ahmedabad, IND; 2 Dermatology, Skin and Hair Research and Education Initiative (SHREI), Ahmedabad, IND; 3 Psychiatry, Banner Health, Phoenix, USA; 4 Medicine, Drexel University College of Medicine, Philadelphia, USA; 5 Medicine, Harvard Medical School, Boston, USA; 6 Research, Global Remote Research Program, Saint Paul, USA; 7 Critical Care Medicine, Mayo Clinic, Rochester, USA; 8 Research, WellSpan Health, York, USA

**Keywords:** body dysmorphia, eternal youth, obsession, aesthetics, dorian gray

## Abstract

The concept of the "Dorian Gray Trait," inspired by Oscar Wilde's renowned literary work, delves into the intricate interplay between self-perception, the fear of aging, and the pursuit of eternal youth. This article explores the psychological dimensions of this trait, wherein individuals harbor an intense preoccupation with maintaining their youthful appearance, often at the cost of their overall well-being. By examining the underlying factors that drive this phenomenon, including societal pressures and personal anxieties, we aim to shed light on the broader implications for mental health and self-image.

## Editorial

The concept “Dorian Gray syndrome” (DGS) was first used in the year 2000. In popular usage, the term is used metaphorically to refer to individuals who strive to maintain an outward appearance of youth and beauty at all costs. However, it is essential to note that this term is not recognized within the medical or psychiatric community as a formal diagnosis or condition.

A 2001 article outlines the defining characteristics of the Dorian Gray syndrome: A) Excessive preoccupation with one’s outward appearance. B) Imaginary or minimal defects in external morphology responded to in the form of embarrassment and social reclusiveness. C) A strong desire to preserve one’s youthfulness in order not to grow older [1].

Dorian Gray syndrome may be considered a variant of body dysmorphic disorder (BDD) due to the similarity in characteristics between the two conditions. Individuals with both conditions exhibit excessive concern and preoccupation with their physical appearance, often focusing on perceived flaws or imperfections. They may engage in repetitive and compulsive behaviors to alleviate their distress such as seeking cosmetic interventions or constantly checking their appearance. The underlying psychological factors, including low self-esteem and distorted self-image, are also shared between Dorian Gray syndrome and BDD [2].

Currently, there are no available data on the prevalence of DGS. However, the estimated prevalence of BDD in the general population is about 2%. The prevalence significantly increases in dermatological settings to up to 11% [3].

The eponymous lead character of Oscar Wilde’s novel “The Picture of Dorian Gray,” keeps his youthful appearance while his portrait ages. Dorian Gray, an exceptionally beautiful man, makes a wish that his newly painted portrait takes the brunt of time while his real self remains unmarred by the ravages of age and circumstance. This wish comes true. He goes to great lengths to hide the painting, which not only shows him advancing in age but also reflects any selfish or immoral act with a dramatically accelerated deterioration (“The quivering ardent sunlight showed him the lines of cruelty round the mouth as clearly as if he had been looking into a mirror after he had done some dreadful thing”). The novel was written at a time when views of hedonism, art, and aesthetics were undergoing a sea change. The victory of self-indulgence and personal pleasure over morality would be frowned upon even now, but the idea of wanting to preserve one’s beauty has surely been delinked with ethicality.

A 45-year-old wife of a businessman visited the dermatology clinic for her routine neurotoxin shots. In her designer outfit, sipping on infused water, she asked about any new aesthetic procedures that may have been introduced in the clinic. She had recently attended a gathering of older adults and was always afraid of turning ‘fat and wrinkly’ like them. Her expectation was that since she was making a lot of efforts like eating gluten-free food, drinking green tea, taking whey protein, working out, and using the most expensive skin care products, she would always look young. A quick probing into her lifestyle revealed an irregular way of life, erratic binging on desserts, frequent partying involving alcohol and hookah (water pipe), and complete sedentariness apart from occasional workouts. A lecture on the importance of a healthy lifestyle was met with an incredulous stare. “Why do you think I visit you, doctor? You must fix me and keep me looking young. I am afraid if I don’t keep up with my high society lifestyle, my husband will leave me.”

We would like to propose a term, ‘Dorian Gray trait,’ for such cases. The proposed Dorian Gray trait is an obsession with a youthful appearance and compulsive use of aesthetic and lifestyle trends to mute or mask signs of aging while circumventing a rational healthy way of life. There is a fear of ‘looking’ old, rather than growing old. A typical patient is likely to be middle-aged, educated, of a middle or higher socioeconomic class, heavily using social media, and having subconscious insecurities about his or her appearance or relationships. There is an attraction for a quick fix (read ‘lunchtime’), instant gratification aesthetic treatments. Exercise is only done for weight loss, not for fitness. A victim of diet fads, this patient spends a lot of money following whatever lifestyle trend is in vogue on social media. Our patient associates all self-worth with her youthful appearance. Although mentioned as an afterthought, the apprehension of her husband, who supported her lavish lifestyle and aesthetic treatments, leaving her due to her aging appearance, appeared quite genuine.

The quest for eternal youth is not a twenty-first-century phenomenon. It is probably as old as civilization. Stories of beautiful women using all kinds of materials, ranging from bizarre to outright toxic, to enhance their beauty are found in cultures around the world. The Fountain of Youth and the Philosophers’ Stone, both purported as means of eternal youth and immortality, have inspired art and literature over centuries. It seems that the fountain of youth today is aesthetic medicine and surgery. Most traditional cultures embrace age, older adults are respected for their experience and wisdom. However, some modern cultures associate age with illness, even considering it a disease that needs ‘treatment.’ Modern cosmetic industry and media glamorize youth and reinforce this belief, hence the focus on ‘anti-aging’ as opposed to ‘healthy aging.’

In the age of social media and selfies, our exposure to our own image is very frequent. The urge to show the best possible appearance of one’s self is natural. But software-modified and beauty-filtered images can show us how we might look with a few alterations like higher cheekbones, fuller lips, or trimmer jawlines, and patients are known to demand aesthetic procedures based on this ‘selfie dysmorphia.’ The rapid progress and widened reach of aesthetic treatments have made it possible for even fantastical expectations regarding appearance to be fulfilled. Baldness is abated, waistlines are slimmed, and wrinkles are erased. Treatments have moved from the realm of need to want. Many middle-aged celebrities flaunt their real, treatment-altered, or morphed youthful looks, which generate demand among the general population, much like the trickledown effect seen in fashion. Earlier considered the bastion of actors and models, aesthetic medicine has been rendered accessible to the wider population at an economical rate.

The Internet is heavily influencing patients’ attitudes towards aesthetic treatments [4]. Patients as young as 16 or 17 are asking for anti-aging treatments [5]. Although our patient is a female, with the rising focus on cosmetics and treatments for males, there will surely be a blurring of this gender typecasting. Statistics from the American Society for Aesthetic Plastic Surgeons (ASAPS) indicated a notable surge of around 55% in plastic surgery procedures among men from 1997 to 2018 [6]. In fact, the primary case described in the flagship paper on the ‘Dorian Gray syndrome’ is a male, as is the hero of the novel.

Dorian Gray pursues personal gratification and indulgences that ultimately lead to great suffering, unhappiness, and death. Today’s indulgences may be fast food, sedentariness, substance abuse, and overuse of social media. Maintaining healthy behaviors throughout life is a slow and tedious mode of slowing down the aging process but is not alluring enough for a patient having the Dorian Gray trait.

Wilde professes “who, that knew anything about life, would surrender the chance of remaining always young?” at the same time confessing that “To get back my youth I would do anything in the world, except take exercise or get up early.”

If this remarkable fantasy were to become a reality, there would be no dearth of takers for it.
